# Pathological complete response after neoadjuvant immunotherapy combined with chemotherapy in pediatric rectal carcinoma: A case report

**DOI:** 10.3389/fimmu.2022.1036181

**Published:** 2022-12-05

**Authors:** Xiaomeng Cao, Jianwei Luo, Baoyin Zhao, Hongjiang Fu, Wendi Kang

**Affiliations:** ^1^ The First Clinical Medical College of Gansu University of Chinese Medicine, Lanzhou, Gansu, China; ^2^ Department of General Surgery, The 940th Hospital of Joint Logistics Support Force of Chinese People’s Liberation Army, Lanzhou, Gansu, China; ^3^ Department of Radiology, Hunan Cancer Hospital, Changsha, Hunan, China; ^4^ Hulunbuir Clinical Medical College, Inner Mongolia University for Nationalities, Hulunbuir, Inner Mongolia Autonomous Region, China; ^5^ Department of Interventional Therapy, National Cancer Center/National Clinical Research Center for Cancer/Cancer Hospital, Chinese Academy of Medical Sciences and Peking Union Medical College, Beijing, China

**Keywords:** pediatric colorectal cancer, pathological complete response, neoadjuvant immunotherapy, microsatellite instability-high, immune checkpoint inhibitors

## Abstract

**Background:**

Pediatric colorectal carcinoma (PCRC) is a rare non-embryonal tumor with an incidence of 0.1% to 1% of adults. Immune checkpoint inhibitors (ICIs) targeting programmed death-1 (PD-1) have shown significant efficacy in defective mismatch repair/Microsatellite instability-high (dMMR/MSI-H) metastatic CRC (mCRC). Although several studies have reported neoadjuvant immunotherapy (NIT) in MSI-H/dMMR non-mCRC patients, not all patients achieved pathological complete remission (pCR). There are differences between PCRC and adult colorectal carcinoma (CRC), and the role of NIT in PCRC remains to be further defined.

**Case presentation:**

We report the case of a 12-year-old child who was admitted to the hospital with abdominal pain and vomiting for more than 3 months. The child’s diagnosis was difficult and complex. He was initially diagnosed with intestinal obstruction, eventually diagnosed with a rare PCRC and identified as locally advanced colorectal cancer (LACRC) with genetic sequencing results showing MSI-H. After a thorough evaluation by clinicians, he received 4 cycles of Camrelizumab (anti-PD-1 antibody) + CapeOx (capecitabine and oxaliplatin) NIT combination chemotherapy. Repeat imaging and all tumor markers were unremarkable, and R0 resection was achieved. Postoperative pathology showed a tumor regression grade (TRG) of 0 grade determined as pCR. Postoperative review has not shown any recurrence or metastasis to date and the prognosis is good.

**Conclusion:**

PCRC should improve the diagnostic efficiency to prevent misdiagnosis and miss the best time for treatment. NIT and or chemotherapy can be a reasonable and effective treatment option for dMMR/MSI-H locally advanced PCRC. Our report provides some support and evidence for neoadjuvant immunotherapy for locally advanced PCRC, while highlighting the importance of preoperative detection of microsatellite status for locally advanced PCRC.

## Introduction

Colorectal cancer (CRC) has the third highest incidence rate and is the second leading cause of cancer death worldwide (1). Pediatric colorectal carcinoma (PCRC) is a rare non-embryonal tumor with an incidence of 0.1% to 1% of adults and accounts for 1% of all childhood malignancies (2, 3). In recent years, the lack of appropriate screening mechanisms in children and young adults has led to a yearly increase in their incidence (4). The clinical presentation of PCRC is atypical and significantly less prevalent than in adults, so patients are clinically staged late at the time of diagnosis. The tissue types of PCRC are mostly mucinous adenocarcinoma and signet-ring cell carcinoma, the percentage of PCRC mucinous adenocarcinoma (including signet-ring carcinoma) is at least twice as high as that of adults, generally up to 45%-50%, which results in a worse overall prognosis of PCRC (3, 5).

Although neoadjuvant chemotherapy reduces the local recurrence rate for patients with LACRC, it is not effective in improving long-term patient survival (6). In recent years, ICIs have become an effective treatment for various cancers (7). ICIs regulate the interaction between T cells, antigen-presenting cells, and tumor cells to help release the suppressed immune response (8). ICIs can also provide improved treatment for CRC and may become an effective treatment for patients with dMMR/MSI-H mCRC (9, 10), a key determinant of the effectiveness of ICIs treatment is the patient’s MSI-H status rather than the specific type of cancer (11).

We report a rare case of 12-year-old PCRC combined with MSI-H, who was initially diagnosed with intestinal obstruction and had a complex and difficult diagnostic and treatment history. He achieved PCR after receiving NIT combined with neoadjuvant chemotherapy, highlighting the positive impact of preoperative NIT and chemotherapy on PCRC, as well as providing support for future neoadjuvant combination therapy for locally advanced PCRC.

## Case description

**Figure 1:**
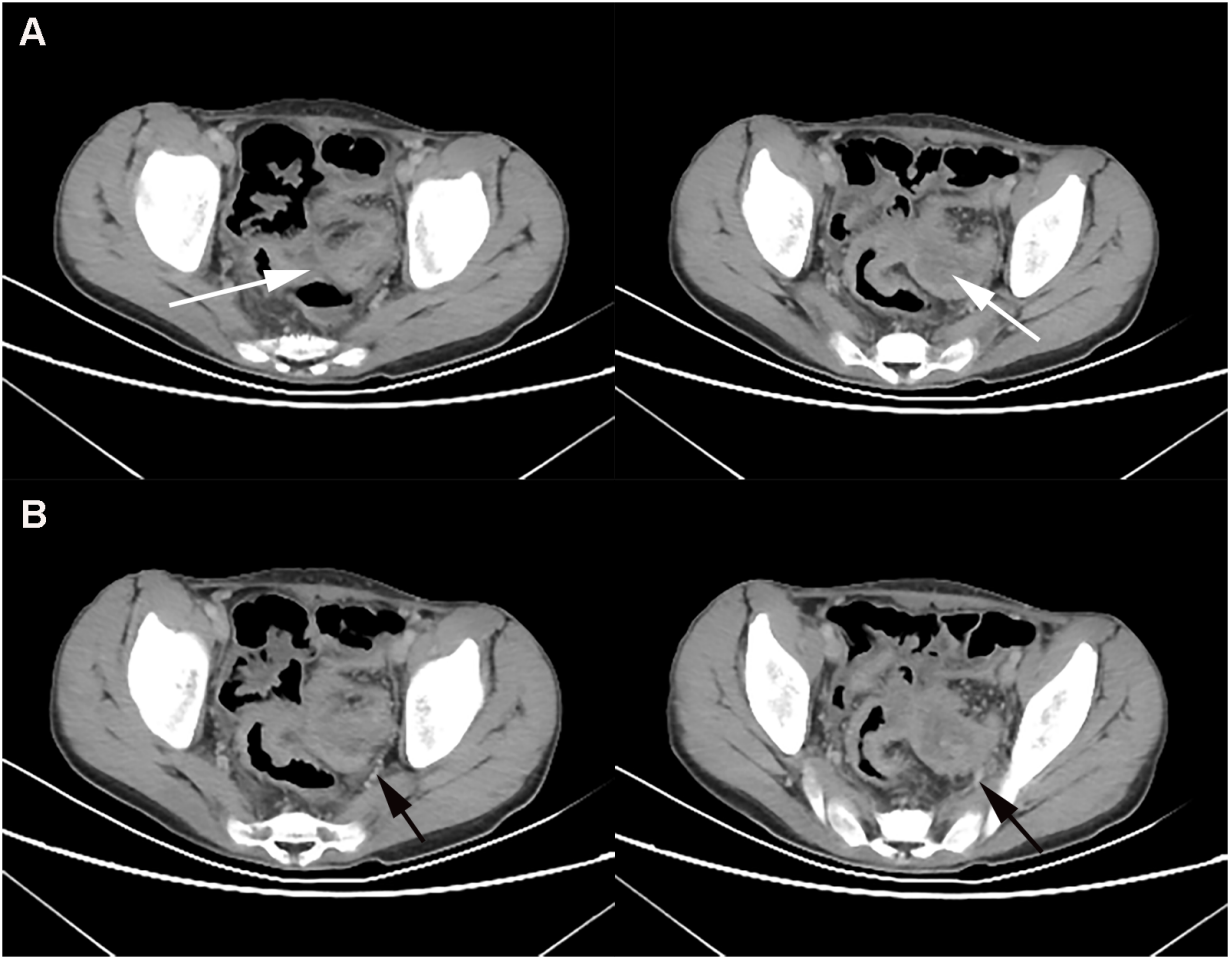
At baseline (November 2021), contrast-enhanced CT showed thickening of the wall of the upper rectum and part of the sigmoid colon with significant narrowing of the intestinal lumen **(A)**, and the lesion was closely associated with the left iliac vessels **(B)**.

A 12-year-old boy presented to our hospital on November 17, 2020 with intermittent abdominal pain, bloating and occasional vomiting for three months. He had diarrhea 3-4 times a day for the last 6 months and lost 7 kg. He underwent an abdominal CT examination at an outside hospital on October 6, 2020, which showed rectal wall thickening, intestinal wall edema, and partial intestinal interstitial encapsulated abscess formation, which was considered inflammatory bowel disease combined with pelvic effusion. Symptoms were slightly relieved after symptomatic treatment was given. The child’s abdominal pain was significantly worse from November 10, 2020, with intermittent pain of longer duration. On November 17, 2020, he was admitted to our hospital for general surgery for abdominal pain. The next day, the child underwent a whole abdominal contrast-enhanced CT scan, which showed segmental thickening of the superior rectal and sigmoid walls with a corresponding narrowing of the intestinal lumen (**Figure 1A**). The lesion was closely associated with the left internal iliac vessel and its branches (**Figure 1B**).

**Figure 2:**
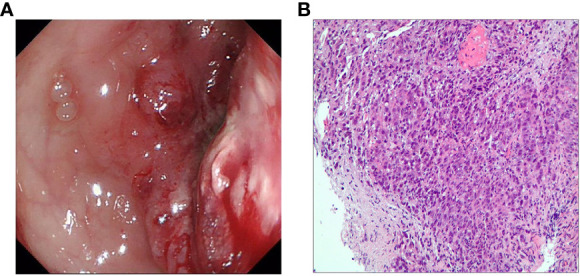
**(A)** Colonoscopy shows a lesion protruding into the intestinal lumen with combined intestinal stricture. **(B)** Preoperative hematoxylin and eosin staining for the diagnosis of poorly differentiated adenocarcinoma of the rectum.

After admission, the child was diagnosed with intestinal obstruction based on history and various examinations, and did not improve after one week of conservative treatment. Colonoscopy showed an irregular mass protruding from the rectum at 12 cm from the anus, with a congested and eroded surface, a lot of necrotic tissue, easy bleeding when touched, and a narrowed circumferential intestinal lumen with resistance to enter the scope, suggesting rectal occupation and stenosis (**Figure 2A**). On December 3, 2020, surgeons performed an exploratory laparotomy to clarify the diagnosis. Intraoperatively, thickening of the rectal wall was seen, which was fixed to the left internal iliac vessels and surrounding tissues and could not be pushed, so a left colostomy and abdominal drainage was performed. Intraoperative histopathology was taken to diagnose a poorly differentiated adenocarcinoma of the rectum (**Figure 2B**), and immunohistochemical results showed Ki67 (index ≈ 70%), TP53 (mutant type), and the genetic test done by the patient at the company showed MSI-H. On December 7, 2020, the pelvic CT-enhanced examination showed segmental thickening of the upper rectal and sigmoid canal walls with soft tissue mass formation and narrowing of the corresponding intestinal lumen, which was consistent with the manifestation of rectal cancer when combined with pathology.

Based on the child’s diagnosis of MS-H locally advanced PCRC, clinicians ultimately decided to treat him with NIT in combination with chemotherapy. He was admitted to our general surgery department on January 5, February 1 and March 3, 2021, and completed 3 cycles of chemotherapy with Camrelizumab + CapeOx (capecitabine and oxaliplatin) in the absence of significant myelosuppression. Camrelizumab regimen was 200 mg every three weeks, oxaliplatin regimen was 130 mg/m^2^ and capecitabine regimen was 1000 mg/m^2^. CEA: 1.01ng/mL, CA-199: 22.0 U/mL on January 6, 2021. CEA: 0.71ng/mL, CA-199: 8.0 U/mL on February 2, 2021. CEA: 0.76ng/mL, CA-199: 6.1 U/mL on March 4, 2021.

**Figure 3:**
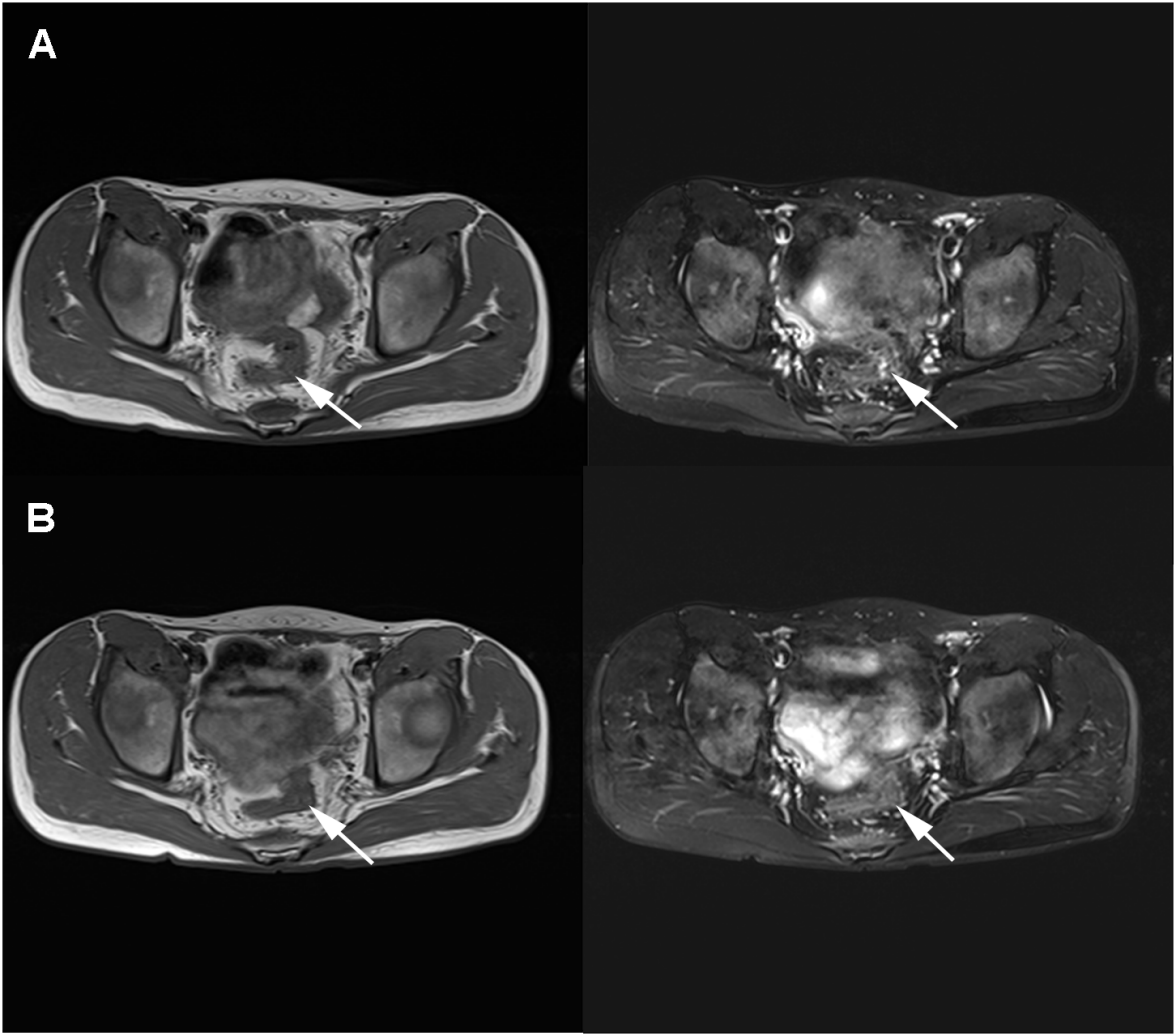
MRI review in March 2021 after neoadjuvant immunotherapy combined with chemotherapy showed a significantly smaller lesion than before **(A)**. MRI review in April 2021 showed a further smaller lesion than before with no significant thickening **(B)**.

**Figure 4:**
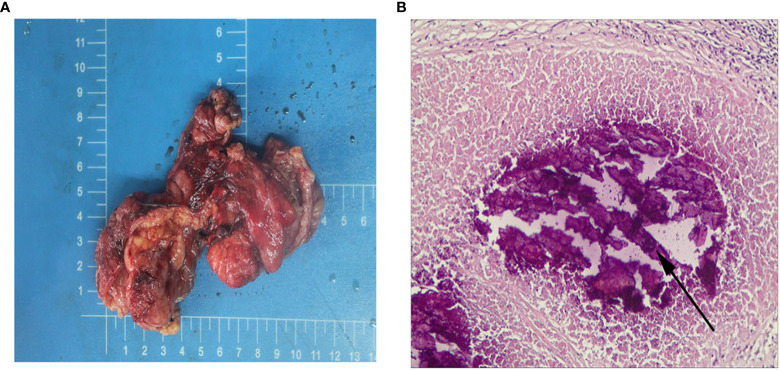
**(A)** Postoperative general. **(B)** Postoperative hematoxylin and eosin stain, the arrows indicate necrosis, original magnification × 200.

**Figure 5:**
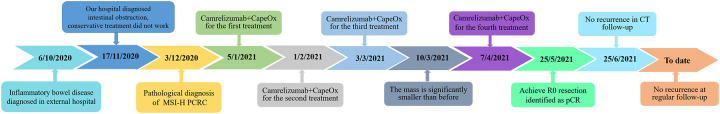
Timeline of the patient’s diagnosis and treatments.

After the child underwent three cycles of neoadjuvant therapy, the pelvic MRI on March 10, 2021 showed significant improvement in the thickening of the upper rectal and sigmoid walls compared to the previous image on December 7, 2020 (**Figure 3A**). The pelvic MRI scan on April 7, 2021 showed further improvement in the upper rectal and sigmoid walls compared to the previous one (**Figure 3B**). No significant masses were seen on imaging review, and the regimen was continued for a fourth cycle of treatment. The child was admitted to our anorectal surgery department on May 14, 2021 with CEA: 0.70ng/mL, CA-199: 4.2 U/mL and no contraindication to surgery. On May 25, 2021, he underwent a colostomy and partial rectal resection under general anesthesia (**Figure 4A**). Postoperative pathological diagnosis showed focal degeneration, necrosis and calcium salt deposition (**Figure 4B**), and positive CKp staining of focal necrotic material was seen in the lymph nodes (7/8), which was determined to be pCR with TRG 0 grade after treatment. He was admitted to our anorectal surgery department on June 22, 2021 for review and further treatment, CEA: 0.78ng/mL, CA-199: 4.2 U/mL. On June 25, 2021, an abdominal enhanced CT showed no significant abnormalities in the operative area. No significant abnormalities were seen on the CT scan of the chest. All tests showed no tumor metastasis or recurrence. The timeline of the patient is summarized in **Figure 5**. There were minimal side effects after immunotherapy and chemotherapy, and he was discharged in good condition. He was regularly reviewed every three months at our outpatient clinic and no recurrence was observed.

## Discussion

PCRC is a rare non-embryonal tumor. Abdominal pain and vomiting are the main clinical symptoms of the onset, which lack characteristics. Because of the low incidence and atypical symptoms, it is difficult for children to accurately describe the symptoms in their medical history, and it is easy to be misdiagnosed as colitis, intussusception, etc. at the early stage; it is easily ignored by non-oncology specialists, resulting in delayed diagnosis and treatment, and the misdiagnosis rate of PCRC is as high as 90% (12). The prognosis of PCRC is worse than that of adults, which is related to the difficulty of early detection, failure to perform timely screening colonoscopy, aggressive tumor behavior and distinct histologic and molecular presentations in children and adolescents with colorectal cancer (13). It has been found that MSI is about twice as common in PCRC as in adult CRC, which may be one of the reasons for the different pathogenesis and prognosis of sporadic cases in children compared with adults (14). In terms of clinical treatment, no other studies on PCRC treatment have been reported worldwide, except for one prospective study on surgery and radiotherapy regimens (15). To date, no other studies on PCRC treatment have been reported, and the treatment of children is still mainly based on adult treatment guidelines (16). The main treatment modalities include surgery, adjuvant chemoradiotherapy and targeted therapy.

dMMR/MSI-H is a promising biomarker for PD-1 inhibitor response that is present in 12-15% of all CRC patients, but only 2% of rectal cancer patients are MSI-H (17). The principle of NIT is to induce T-cell expansion and lead to reduced T-cell depletion through elevated levels of endogenous tumor antigens in the tumor, while killing tumor cells, promoting preoperative tumor downstaging and improving resection and pathological remission rates (18). ICIs are highly effective treatments for patients with mCRC. Meanwhile a part of clinical trials have been started for NIT in dMMR/MSI-H non-mCRC patients with good clinical results, and some of them are still continuing. The single-arm NICHE study from the Netherlands in 2020 (19), an important study of NIT, enrolled 40 patients with stage I-III colon cancer and showed that the dual immune neoadjuvant nivolumab + ipilimumab regimen is appropriate for NIT in patients with dMMR non-mCRC. The NICHE study pioneered the use of NIT in CRC, offering hope for MSI -H/dMMR LACRC patients offers hope. Several other recently published studies evaluating the role of ICIs in LACRC have shown high rates of achieving pCP with NIT in patients with locally advanced dMMR/MSI-H CRC (20–24). To be sure, it is remarkable that consecutive series of studies achieved complete or near pathological response to NIT treatment in all patients. Eventually, larger prospective studies are also needed to confirm the generalizability of these findings and their application as a standard of care.

Although several cases and clinical trials have reported the surprising efficacy of NIT and or neoadjuvant chemotherapy in LACRC (20, 22, 25, 26). To our knowledge, there is no report of achieving cPR after NIT combined with neoadjuvant chemotherapy in PCRC. The present case is the first report of MSI-H PCRC achieving cPR after NIT combined with neoadjuvant chemotherapy treatment. The diagnosis of this case was difficult and complex, with an initial diagnosis of intestinal obstruction, ineffective treatment with symptomatic conservative therapy, and a final intraoperative pathological diagnosis of rare PCRC with genetic sequencing results showing MSI-H. Therefore, clinicians chose to treat him with 4 cycles of NIT combined with chemotherapy Camrelizumab + CapeOx regimen. After the completion of 4 cycles of treatment, tumor markers returned to normal, no significant tumor was seen on imaging, and postoperative cPR was determined to be R0 resection, indicating that this child benefited from neoadjuvant combination therapy. This case is partially instructive for the treatment of dMMR/MSI-H PCRC. First, MSI detection is important for locally advanced PCRC, and NIT combined with neoadjuvant chemotherapy can achieve pCR for this type of PCRC. Second, children with PCRC are young, and considering the toxic effects of chemotherapeutic drugs and the existing studies emphasizing the powerful efficacy of immunotherapy, cPR may also be achieved preoperatively with NIT only, thus reducing the multiple chemotherapeutic the toxic effects of multiple chemotherapy drugs on the children.

PCRC is highly malignant and aggressive, so special attention should be paid to those children with a familial tendency. It is important to be alert to symptoms such as blood in the stool and abdominal pain in children to avoid misdiagnosis and delay in optimal treatment. PCRC still has surgical resection of the tumor as the primary treatment and goal, but early diagnosis and multi-path and multi-method treatment, especially NIT for dMMR/MSI-H locally advanced PCRC, can improve survival. The goals of NIT are to improve tumor response, induce complete pathologic response, simplify surgery, and cure patients, and such goals also apply to PCRC. Therefore, we recommend that PCRC should be guided by risk level classification and molecular typing, leading to the selection of neoadjuvant therapy. For dMMR/MSI-H locally advanced PCRC the combination of NIT and or neoadjuvant radiotherapy is preferred and may be a reasonable choice and promising treatment strategy.

## Conclusion

In summary, PCRC should improve the diagnostic efficiency to prevent misdiagnosis and miss the best time for treatment. NIT and or chemotherapy can be a reasonable and effective treatment option for dMMR/MSI-H locally advanced PCRC. Our report provides some support and evidence for neoadjuvant immunotherapy for locally advanced PCRC, while highlighting the importance of preoperative detection of microsatellite status for locally advanced PCRC. Of course, more research is needed to further confirm the effectiveness of this strategy.

## Data availability statement

The original contributions presented in the study are included in the article/supplementary material. Further inquiries can be directed to the corresponding author.

## Ethics statement

The studies involving human participants were reviewed and approved by The 940th Hospital of Joint Logistics Support Force of Chinese People’s Liberation Army Ethics Committee. Written informed consent to participate in this study was provided by the participants’ legal guardian/next of kin. Written informed consent was obtained from the minor(s)’ legal guardian/next of kin for the publication of any potentially identifiable images or data included in this article.

## Author contributions

Writing original draft: XC. Supervision: WK. All authors participated in the revision of the manuscript. All authors have read and agreed to the published version of the manuscript. All authors contributed to the article and approved the submitted version.

## Conflict of interest

The authors declare that the research was conducted in the absence of any commercial or financial relationships that could be construed as a potential conflict of interest.

## Publisher’s note

All claims expressed in this article are solely those of the authors and do not necessarily represent those of their affiliated organizations, or those of the publisher, the editors and the reviewers. Any product that may be evaluated in this article, or claim that may be made by its manufacturer, is not guaranteed or endorsed by the publisher.
